# Combining Clinical Parameters and Acute Tubular Injury Grading Is Superior in Predicting the Prognosis of Deceased-Donor Kidney Transplantation: A 7-Year Observational Study

**DOI:** 10.3389/fimmu.2022.912749

**Published:** 2022-06-30

**Authors:** Jiali Wang, Jinqi Liu, Wenrui Wu, Shicong Yang, Longshan Liu, Qian Fu, Jun Li, Xutao Chen, Ronghai Deng, Chenglin Wu, Sizhe Long, Wujun Zhang, Huanxi Zhang, Haiping Mao, Wenfang Chen

**Affiliations:** ^1^ Department of Nephrology, The First Affiliated Hospital, Sun Yat-sen University, Yuexiu District, Guangzhou, China; ^2^ Department of Pediatrics, Guangzhou Women and Children’s Medical Centre, Guangzhou, China; ^3^ Organ Transplant Center, The First Affiliated Hospital, Sun Yat-sen University, Guangzhou, China; ^4^ Department of Pathology, The First Affiliated Hospital, Sun Yat-sen University, Guangzhou, China; ^5^ Guangdong Provincial Key Laboratory on Organ Donation and Transplant Immunology, Guangzhou, Guangzhou, China; ^6^ Guangdong Provincial International Cooperation Base of Science and Technology (Organ Transplantation), Guangzhou, China; ^7^ Center for Information Technology and Statistics, The First Affiliated Hospital, Sun Yat-sen University, Guangzhou, China

**Keywords:** kidney transplantation, pretransplant biopsy, acute tubular injury, delayed graft function, deceased donor

## Abstract

**Background:**

We developed a pragmatic dichotomous grading criterion to stratify the acute tubular injury (ATI) of deceased-donor kidneys. We intended to verify the predictive value of this criterion for the prognosis of deceased-donor kidney transplantation.

**Methods:**

The allografts with ATI were classified into severe and mild groups. Severe ATI was defined as the presence of extreme and diffuse flattening of the tubular epithelial cells, or denudement of the tubular basement membrane. The clinical delayed graft function (DGF) risk index was calculated based on a regression model for posttransplant DGF using 17 clinical parameters related to donor–recipient characteristics.

**Results:**

A total of 140 recipients were enrolled: 18 severe and 122 mild ATI. Compared with the mild ATI group, the severe ATI group had more donors after cardiac death, higher median donor terminal serum creatinine level (dScr), and longer median cold ischemia time. Severe ATI had a higher DGF rate (55.6% vs 14.6%, p < 0.001), longer DGF recovery time (49.6 vs 26.3 days, p < 0.001), and a lower estimated glomerular filtration rate (eGFR) at 1 month (23.5 vs 54.0 ml/min/1.73 m^2^, p < 0.001), 3 months (40.4 vs 59.0, p = 0.001), and 6 months after transplant (46.8 vs 60.3, p = 0.033). However, there was no significant difference in eGFR at 1 year or beyond, graft, and patient survival. The predictive value of combined dScr with ATI severity for DGF rate and DGF recovery time was superior to that of dScr alone. The predictive value of the combined DGF risk index with ATI severity for DGF was also better than that of the DGF risk index alone; however, the association of the DGF risk index with DGF recovery time was not identified. Chronic lesions including glomerulosclerosis, interstitial fibrosis, arterial intimal fibrosis, and arteriolar hyalinosis were associated with declined posttransplant 1-year eGFR.

**Conclusion:**

Based on our pragmatic dichotomous grading criterion for ATI in a preimplantation biopsy, donor kidneys with severe ATI increased DGF risk, prolonged DGF recovery, and decreased short-term graft function but demonstrated favorable long-term graft function. Our grading method can offer additive valuable information for assessing donor kidneys with acute kidney injury and may act as an effective supplementary index of the Banff criteria.

## Introduction

Kidney transplantation is the best treatment for patients with irreversible chronic kidney failure (1). An increasing number of transplantation candidates and a relative shortage of donor kidneys have resulted in the increasing use of marginal donor kidneys (2–3). However, marginal donor kidneys may be a risk factor for poor allograft outcomes (4–6). Thus, evaluating marginal donor kidneys and determining outcome predictors are important.

Kidneys with acute kidney injury (AKI) from deceased donors are considered marginal donor kidneys (7–10). Serum creatinine is a common clinical indicator of AKI, and the RIFLE (Risk, Injury, Failure, Loss of kidney function, and End-stage kidney disease), AKIN (Acute Kidney Injury Network), and KDIGO (Kidney Disease: Improving Global Outcomes) criteria all use it as a biomarker (11–13). Some studies have indicated that donor kidneys with AKI are associated with an increased incidence of delayed graft function (DGF), but there is no influence on long-term allograft function (14–18). AKI in these studies has typically been defined as an elevated donor terminal serum creatinine level (≥1.7 mg/dl) (14–18). However, using donor terminal creatinine only to assess AKI ignores other factors that cause damage to graft, including ischemia during the process of dying and the damage during organ procurement, transportation, and preservation (19–21). Moreover, terminal donor creatinine level does not distinguish between the influence of the AKI and that of chronic kidney disease. Neither can it exclude thrombosis and extensive infarction. An elevated creatinine level may be associated with reversible kidney injury and does not necessarily indicate there is a structural injury of the kidney (19–23). Donor serum creatinine is not a sensitive indicator and may not necessarily peak at the time of organ procurement, failing to reflect the severity of AKI. Therefore, to assess donor kidney AKI based solely on donor serum creatinine is insufficient. Apart from donor creatinine, some studies have also developed models for predicting short-term and long-term kidney transplantation outcomes based on multiple clinical parameters to assess donor kidney quality, but most of them have not been externally validated and thus cannot be generalized (24). Among them, the Irish 2010 model is a good externally validated model for predicting the incidence of DGF (25, 26), which requires clinical information from both donors and recipients. KDRI/KDPI (kidney donor risk index/ kidney donor profile index) is another reliable graft survival prediction model for donor kidney quality assessment using donor clinical characteristics (27). These multiparameter clinical models may offer more guidance for AKI donor kidney evaluation.

Another method to identify and quantify donor AKI is pretransplantation renal biopsy (28–34). All causes of acute injury to the kidney allograft can be reflected on histopathological examination of a pretransplantation biopsy, which mainly manifests as acute tubular injury (ATI) (19–21, 29, 33). Renal biopsy can also distinguish acute and chronic lesions of the donor kidneys and exclude diffuse thrombosis and extensive infarction. However, a pretransplantation biopsy is not routinely performed at many transplant centers. The results from the previous studies examining the relationship between pathological changes seen on pretransplantation biopsy and graft function after transplantation were contradictory. A multicenter study by Hall et al. (33) revealed no significant associations overall between preimplant biopsy-reported acute tubular necrosis (ATN) and the outcomes of DGF or graft failure. Matignon et al. (31) and Oberbauer et al. (28) both reported that ATI was not associated with DGF. On the other hand, Sulikowski et al. (5) found that biopsy-proven ATI was associated with DGF and primary non-graft function. A possible important reason for these differences in results is the different definitions and pathological descriptions of ATI or ATN. For example, Hall et al. (33) classified all degrees of ATI as ATN, which would dilute the impact of severe ATI. Currently, the international histopathological criteria for donor kidney evaluation are the Banff criteria (35). The grading of ATI/ATN is as follows: mild = epithelial flattening, tubule dilation, nuclear dropout, and loss of brush border; moderate = focal coagulative type necrosis; severe = infarction. Signs of necrosis are essential in moderate-to-severe degrees. It is understandable that the donor kidney Banff criteria are classified in this way because they are primarily used to determine whether a donor kidney should be discarded, and necrosis is one of the important determinants. However, very few of the donor kidneys we have received have met the criteria of moderate to severe. Instead, the vast majority fall in the mild category. The Banff criteria do not allow for more precise grading of ATI, and it is difficult to make prognostic judgments and risk grading of AKI donor kidneys. In addition, donor kidney evaluation often requires urgent and accurate judgment from the pathologist on a rush basis. Therefore, we need to develop refined and pragmatic ATI grading criteria that can be used to predict the short-term and long-term outcomes of kidney transplantation. A systematic review has summarized 16 pathological descriptions of acute renal tubular injury (36). After combining and screening the 16 pathological changes, we finally developed a simple and easy-to-use dichotomous (mild-to-severe) grading criterion for donor allograft ATI. We intend to clarify the discriminative and predictive ability of this grading criterion for the short-term and long-term prognoses by retrospective analysis.

## Methods

### Patients

We retrospectively reviewed the medical records of adult (≥18 years old) kidney transplant recipients who received deceased-donor kidneys, including organ donation after brain death (DBD) and donation after circulatory death (DCD), in which a pretransplantation donor kidney biopsy was performed, at the First Affiliated Hospital of Sun-Yat Sen University from Jan 2012 to June 2017. Recipient and donor demographic, clinical, and pathological data were extracted from the medical records of the follow-up database. These recipients were followed up until 2021. This study was approved by the Institutional Review Board of the hospital, and because of the retrospective nature of the study, the requirement of informed patient consent was waived. No organs from executed prisoners were used.

### Preimplantation Biopsies

Biopsies were performed immediately before implantation by using a 16-gauge biopsy needle. The biopsy was performed at the superior edge of the donor kidneys, and two cores of tissue were obtained. Frozen sections of one core were stained with H&E and examined to determine whether to use or discard the kidney. Multiple frozen sections were also saved for subsequent immunofluorescence stains for immunoglobulin and complements including IgG, IgM, IgA, C3, and C1q. The other core and the rest of the frozen tissue were fixed, paraffin-embedded, and stained with H&E, periodic acid–Schiff (PAS), periodic Schiff-methenamine silver (PASM), and Masson’s trichrome (MASSON). Stained biopsy specimens were examined retrospectively by 2 experienced pathologists for a comprehensive assessment of glomerular, tubular, interstitial, and vascular lesions in the donor kidneys.

### Grouping

**Figure 1:**
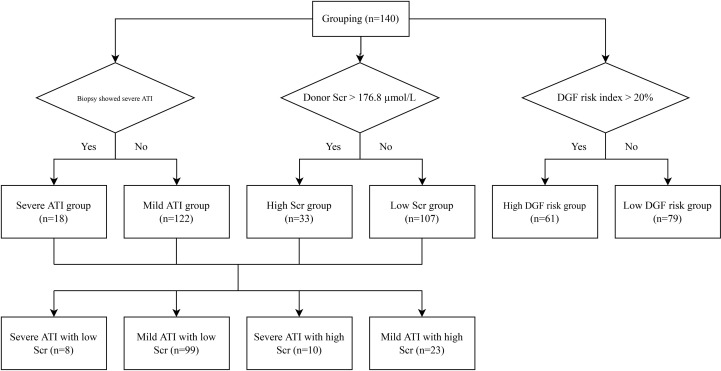
Grouping flowchart. ATI, acute tubular injury; Scr, serum creatinine; DGF, delayed graft function.

**Figure 2:**
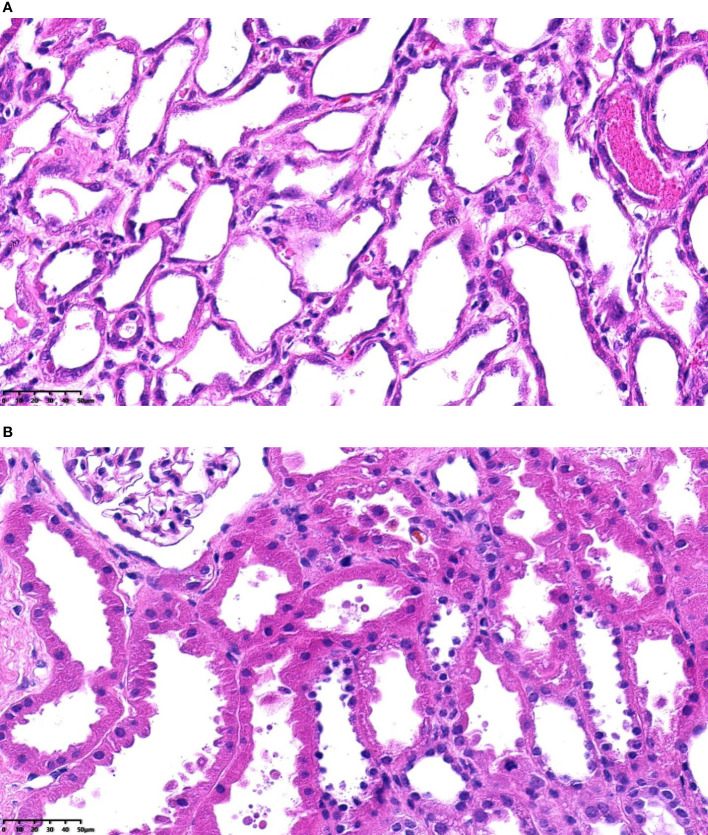
**(A)** Severe acute tubular injury, H&E-stained paraffin sections (magnification, ×400). **(B)** Mild acute tubular injury, H&E-stained paraffin sections (magnification, ×400).

Donor kidneys with tubular coagulative necrosis and parenchymal infarction were excluded from the study. The recipients enrolled were classified into 2 groups based on whether or not the pretransplantation renal biopsy showed severe ATI (**Figure 1**). Severe ATI was defined as the presence of extreme and diffuse (>50%) flattening of the tubular epithelial cells or denudement of tubular basement membrane (TBM) (**Figures 2A, B**). Mild ATI was defined as the absence of severe ATI and no significant effacement of tubular epithelial cells or only mild detachment of brush border. Demographic and clinical characteristics as well as the outcomes were compared between the 2 groups. The chronic kidney injury was assessed using the Remuzzi scoring system (37), including global glomerulosclerosis, interstitial fibrosis, tubular atrophy, arterial intimal fibrosis, and arteriolar hyalinosis.

The patients were divided into high and low donor serum creatinine groups based on donor terminal serum creatinine levels, with a cutoff value of 176.8 µmol/L (2 mg/dl). The high creatinine group was roughly equivalent to stage 2 and 3 AKI according to the KDIGO criteria (13). To assess whether pretransplant renal pathology has additive value in the assessment of the prognosis of AKI donor kidney transplantation compared with the use of serum creatinine alone, the patients were divided into four groups: 1) mild ATI with low terminal serum creatinine, 2) mild ATI with high terminal serum creatinine, 3) severe ATI with low terminal serum creatinine, and 4) severe ATI with high terminal serum creatinine.

Clinical prediction models are also a means to assess the prognosis of AKI donor kidneys, and we have previously validated models published internationally (26), with the Irish 2010 model performing best (25). In the present study, we calculated the predicted risk of DGF occurrence based on the Irish 2010 model by using 17 clinical parameters related to donor–recipient characteristics, including panel reactive antibody (PRA), duration of dialysis, recipient body mass index (BMI), HLA mismatch, cold ischemia time, warm ischemia time, donor terminal creatinine, donor age, donor weight, race, gender, previous transplant, recipient diabetes, recipient pretransplant transfusion, donation after cardiac death, donor history of hypertension, and donor cause of death. The patients were divided into high- and low-risk groups for DGF with 20% as the cutoff.

### Outcome

Measures of short-term posttransplantation allograft function were the occurrence of DGF, DGF recovery time, and estimated glomerular filtration rate (eGFR) at 3 and 6 months. Long-term graft function was evaluated by eGFR at 1–3 years after transplantation and death censored graft survival and patient survival. Glomerular filtration rate was estimated using the Modification of Diet in Renal Disease (MDRD) Study formula (38). If the patient died, the eGFR was counted as censored. If the patient lost his/her graft function, the eGFR was labeled as 0 in the calculation. DGF was defined as the need for dialysis within the first week after transplantation. DGF recovery time was defined as the time from posttransplantation day 1 to the day the recipient achieved a stable eGFR (eGFR did not change by more than 10% in the following week) (39), which was demonstrated to be associated with poor prognosis (rejection, infection, loss of graft function, and death) in our previous study.

We also collected posttransplant indication renal allograft biopsy results from the patients with severe ATI to observe the pathological change of renal tubular injury at different times after surgery to demonstrate AKI recovery.

### Statistical Analysis

Continuous data with normal distribution were presented as mean ± SD and compared using Student’s t-test. Continuous data without a normal distribution were expressed as median (interquartile range [IQR]) and compared using the Mann–Whitney U test. Categorical data were reported as counts and percentages and compared using a chi-square test or Fisher’s exact test. Comparison of continuous data among multiple groups was performed using one-way ANOVA or the Kruskal–Wallis test. Logistic regression and linear regression were used for univariate and multivariate analyses. The likelihood-ratio test was used for the comparison of model performance. All statistical tests were 2-sided, and values of p < 0.05 were considered to indicate statistical significance. Statistical analyses were performed with R software (version 3.6.1).

## Results

### Donor and Recipient Characteristics

A total of 140 recipients were included in the analysis with a median follow-up time of 4.0 (IQR 3.1–5.0) years. Eighteen of the recipients received donor kidneys with severe ATI identified on pretransplantation biopsy, and 122 received kidneys with mild ATI (**Table 1**). Pretransplant PRA was absent in all recipients. Recipient characteristics were similar between the severe and mild ATI groups. With respect to donor characteristics, as compared with the mild ATI group, the severe ATI group had more DCD donors (77.8% vs 29.5%, p < 0.001), higher median donor terminal serum creatinine level (232 vs 94 µmol/L, p = 0.001), and longer median cold ischemia time (15 vs 10 h, p < 0.001).

**Table 1:** Demographics and characteristics of donors and recipients.

Characteristic	All (140)	Mild ATI (122)	Severe ATI (18)	p-Value
**Recipient**				
Age, years	42.8 ± 12.9	42.9 ± 13.1	41.6 ± 11.7	0.650
Male	102 (72.9%)	91 (74.6%)	11 (61.1%)	0.260
BMI, kg/m^2^	21.5 ± 3.3	21.4 ± 3.2	21.8 ± 3.7	0.683
History of diabetes	19 (13.6%)	19 (15.6%)	0	0.132
Cause of ESRD				0.360
Chronic glomerulitis	125 (89.3%)	110 (90.2%)	15 (83.3%)	
Diabetic nephropathy	3 (2.1%)	3 (2.5%)	0	
IgA nephropathy	4 (2.9%)	3 (2.5%)	1 (5.6%)	
Others	8 (5.7%)	6 (4.9%)	2 (11.1%)	
Induction				0.310
ATG	119 (85.0%)	102 (83.6%)	17 (94.4%)	
Basiliximab	21 (15.0%)	20 (16.4%)	1 (5.56%)	
Anti-proliferative agent				0.842
EC-MPS	94 (67.1%)	81 (66.4%)	13 (72.2%)	
MMF	44 (31.4%)	39 (32.0%)	5 (27.8%)	
Others	2 (1.43%)	2 (1.64%)	0 (0.00%)	
Calcineurin inhibitors				1.000
Tacrolimus	138 (98.6%)	120 (98.4%)	18 (100%)	
Cyclosporine	2 (1.43%)	2 (1.64%)	0	
**Donor**				
Age, years	32.5 ± 15.1	32.8 ± 14.6	30.8 ± 18.4	0.676
Male	106 (75.7%)	93 (76.2%)	13 (72.2%)	0.770
BMI, kg/m^2^	22.2 ± 4.4	22.3 ± 4.6	21.6 ± 3.0	0.425
History of hypertension	16 (11.4%)	16 (13.1%)	0	0.225
History of diabetes	3 (2.1%)	3 (2.5%)	0	1.000
Terminal serum creatinine, μmol/L	95[75, 165]	94[74, 149]	232[84, 447]	0.001^**^
DCD (%)	50 (35.7%)	36 (29.5%)	14 (77.8%)	<0.001^***^
Cold ischemia time, h	11[7, 14]	10[7, 13]	15[11, 21]	<0.001^***^

ATI, acute tubular injury; ATG, anti-thymocyte globulin; BMI, body mass index; DCD, donation after circulatory death; EC-MPS, enteric-coated mycophenolate sodium; ESRD, end-stage renal disease; MMF, mycophenolate mofetil.

^*^<0.05; ^**^<0.01; ^***^<0.001.

### Impact on Outcomes

Compared with the grafts with mild ATI, those with severe ATI had a significantly higher DGF rate, longer DGF recovery time, and a lower eGFR at 1, 3, and 6 months after transplant (**Table 2**). However, there was no significant difference in 1-year, 2-year, and 3-year eGFR between severe ATI and mild ATI groups. In contrast, higher donor serum creatinine was not associated with the DGF recovery time. The incidence of DGF was significantly higher in the group with high calculated risk group compared with low calculated risk, but the differences in DGF recovery time and in eGFR at different posttransplant time points were not statistically significant between the two groups (**Table 3**). No significant difference was identified in 3-year death-censored graft survival or patient survival between the two groups regardless of the method of grouping.

**Table 2:** Comparison of posttransplant allograft outcome based on the severity of ATI and the level of donor terminal serum creatinine.

	Mild ATI (122)	Severe ATI (18)	p-Value	Low donor creatinine (107)	High donor creatinine (33)	p-Value
**DGF, n (%)**	18 (14.8)	10 (55.6)	<0.001^***^	15 (14.0)	13 (39.4)	0.003^**^
**DGF recovery time (days)^#^**	26.3 (17.0)	49.6 (10.5)	<0.001^***^	36.2 (18.6)	33.7 (19.3)	0.759
**eGFR at 1 month**	54.0 (21.4)	23.5 (15.1)	<0.001^***^	53.8 (23.7)	38.3 (16.5)	<0.001^***^
**eGFR at 3 months**	59.0 (20.6)	40.4 (19.9)	0.001^**^	59.0 (21.4)	48.6 (19.4)	0.011^**^
**eGFR at 6 months**	60.3 (20.7)	46.8 (23.8)	0.033^*^	60.5 (21.3)	52.1 (21.3)	0.053
**eGFR at 1 year**	62.0 (21.5)	57.0 (25.1)	0.435	63.1 (22.0)	55.7 (21.5)	0.090
**eGFR at 2 years**	61.1 (21.8)	53.3 (21.2)	0.197	61.5 (22.5)	55.4 (18.4)	0.145
**eGFR at 3 years**	60.3 (24.3)	53.4 (23.7)	0.358	60.7 (24.9)	55.1 (21.0)	0.276
**eGFR at 4 years**	62.2 (26.3)	55.1 (24.7)	0.439	63.1 (26.7)	52.9 (21.5)	0.134
**DCGS at 7 years (%)**	96.2 (92.6–100.0)	94.4 (84.4–100.0)	0.560	95.8 (91.9–100.0)	96.2 (89.0–100.0)	0.899
**Patient survival at 7 years (%)**	94.4 (89.4–99.6)	94.1 (83.6–100.0)	0.577	93.5 (87.8–99.6)	97.0 (91.3–100.0)	0.765

The unit of eGFR is ml/min/1.73 m^2^.

ATI, acute tubular injury; DCGS, death-censored graft survival; eGFR, estimated glomerular filtration rate; DGF, delayed graft function.

^#^DGF recovery time was only evaluated in grafts developing DGF.

^*^<0.05; ^**^<0.01; ^***^<0.001.

**Table 3:** Comparison of posttransplant allograft outcome based on the calculated DGF risk derived from Irish 2010 model.

	Low calculated risk (79)	High calculated risk (61)	p-Value
**Calculated risk**	0.08 [0.05; 0.11]	0.30 [0.26; 0.48]	<0.001^***^
**DGF, n (%)**	4 (5.1)	24 (39.3)	<0.001^***^
**DGF recovery time (days)^#^**	27.7 (17.7)	36.1 (18.9)	0.505
**eGFR at 1 month**	53.2 (22.4)	45.9 (23.5)	0.067
**eGFR at 3 months**	57.9 (20.6)	54.8 (22.4)	0.394
**eGFR at 6 months**	59.8 (20.4)	56.8 (23.0)	0.423
**eGFR at 1 year**	61.7 (20.8)	60.8 (23.6)	0.824
**eGFR at 2 years**	60.9 (20.8)	59.3 (23.2)	0.673
**eGFR at 3 years**	60.9 (24.1)	58.0 (24.5)	0.526
**eGFR at 4 years**	62.1 (24.7)	60.5 (28.5)	0.791
**DCGS at 7 years (%)**	96.9 (92.7–100.0)	94.8 (89.1–100.0)	0.422
**Patient survival at 7 years (%)**	92.5 (85.2–100.0)	96.6 (92.2–100.0)	0.674

The unit of eGFR is ml/min/1.73 m^2^.

DCGS, death-censored graft survival; eGFR, estimated glomerular filtration rate; DGF, delayed graft function.

^#^DGF recovery time was only evaluated in grafts developing DGF.

^*^<0.05; ^**^<0.01; ^***^<0.001.

Among the grafts with high donor terminal serum creatinine, those with severe ATI had higher DGF risk, longer DGF recovery time, and lower 1-month eGFR, as compared with those with mild ATI (DGF, 70.0% vs 26.1%, p = 0.026; DGF recovery time, 48.3 ± 11.2 vs 16.2 ± 8.6 days, p < 0.001; 1-month eGFR, 22.4 ± 11.5 vs 45.2 ± 13.4 ml/min/1.73 m^2^, p < 0.001) (**Table 4**). Among the grafts with low donor terminal serum creatinine, those with severe ATI had longer DGF recovery time and lower 1-month, 3-month, and 6-month eGFR, as compared with those with mild ATI (DGF recovery time, 52.0 ± 10.6 vs 31.4 ± 18.2 days, p = 0.049; 1-month eGFR, 24.8 ± 19.5 vs 56.1 ± 22.5 ml/min/1.73 m^2^, p = 0.002; 3-month eGFR, 36.5 ± 18.7 vs 60.9 ± 20.7 ml/min/1.73 m^2^, p = 0.007; 6-month eGFR, 40.9 ± 21.1 vs 62.1 ± 20.6 ml/min/1.73 m^2^, p = 0.025). The predictive ability of combined donor creatinine level with ATI severity for DGF was significantly better than that of donor creatinine level alone (likelihood-ratio test, p = 0.004) or that of ATI severity alone (p = 0.037) (**Table 5**). No interaction between ATI severity and donor creatinine level was identified. The predictive ability of combined calculated DGF risk with ATI severity for DGF was also better than that of calculated DGF risk alone (likelihood-ratio test, p = 0.012). ATI severity is considered the mediator variable between the risk factors for ATI (e.g., DCD and cold ischemia time) and allograft outcome; therefore, the risk factors for ATI were excluded from the multivariable analysis.

**Table 4:** Comparison of kidney allograft outcome among different combinations of donor terminal serum creatinine level and ATI severity.

	Mild ATI with low creatinine (99)	Severe ATI with low creatinine (8)	Mild ATI with high creatinine (23)	Severe ATI with high creatinine (10)	Overall p-Value
**DGF, n (%)**	12 (12.1%)	3 (37.5%)	6 (26.1%)	7 (70.0%)^$^	<0.001^***^
**DGF recovery time (days)^#^**	31.4 (18.2)	52.0 (10.6)^&^	16.2 (8.6)	48.3 (11.2)^$^	0.004^**^
**eGFR at 1 month**	56.1 (22.5)	24.8 (19.5)^&^	45.2 (13.4)	22.4 (11.5)^$^	<0.001^***^
**eGFR at 3 months**	60.9 (20.7)	36.5 (18.7)^&^	50.8 (18.6)	43.5 (21.3)	0.001^**^
**eGFR at 6 months**	62.1 (20.6)	40.9 (21.1)^&^	52.4 (19.6)	51.4 (25.8)	0.010^*^
**eGFR at 1 year**	64.0 (21.7)	51.9 (24.0)	53.3 (19.1)	61.1 (26.5)	0.111
**eGFR at 2 years**	62.7 (22.2)	47.7 (23.5)	54.0 (18.7)	59.7 (17.8)	0.134
**eGFR at 3 years**	62.0 (24.6)	45.2 (25.3)	52.4 (21.6)	65.0 (17.3)	0.158
**eGFR at 4 years**	64.4 (26.4)	48.6 (28.5)	48.8 (22.5)	68.1 (5.40)	0.164
**DCGS at 5 years (%)**	96.5 (92.7–100.0)	87.5 (67.3–100.0)	95.0 (85.9–100.0)	100.0	0.516
**Patient survival at 5 years (%)**	93.1 (87.2–99.5)	100.0	100.0	90.0 (73.2–100.0)	0.385

The unit of eGFR is ml/min/1.73 m^2^.

ATI, acute tubular injury; DCGS, death-censored graft survival; DGF, delayed graft function; eGFR, estimated glomerular filtration rate.

^#^DGF recovery time was only evaluated in grafts developing DGF.

^*^<0.05; ^**^<0.01; ^***^<0.001.

^$^Significant compared with mild ATI with high creatinine at a level of 0.05.

^&^Significant compared with mild ATI with low creatinine at a level of 0.05.

**Table 5:** Univariable and multivariable analyses of pathological factors affecting DGF and 1-year eGFR.

Factor	DGF, odds ratio (95% CI, p-value), univariate	DGF, odds ratio (95% CI, p-value), multivariate	DGF recovery time (95% CI, p-value), univariate	1-year eGFR, coefficient (95% CI, p-value), univariate	1-year eGFR, coefficient (95% CI, p-value), multivariate^#^
ATI (severe vs mild)	7.22 (2.53–21.42, p < 0.001) ^***^	5.31 (1.75–16.42, p = 0.003) ^**^	23.22 (10.15 to 36.29, p = 0.001) ^**^	−4.97 (−15.96 to 6.03, p = 0.373)	−6.43 (−16.80 to 3.94, p = 0.222)
Donor terminal serum creatinine (high vs low)	3.99 (1.64–9.76, p = 0.002) ^**^	2.83 (1.07–7.34, p = 0.033) ^*^	−2.43 (−18.54 to 13.69, p = 0.758)	−7.42 (−16.03 to 1.20, p = 0.091)	–
Calculated DGF risk (high vs low)	12.16 (4.32–43.69, p < 0.001) ^***^	–	8.43 (−15.61 to 32.47, p = 0.475)	−0.86 (−8.33 to 6.62, p = 0.821)	–
Glomerulosclerosis (yes or no)	1.42 (0.56–3.44, p = 0.445)	–	–	−10.91 (−19.16 to −2.66, p = 0.010) ^*^	Excluded
Interstitial fibrosis (yes or no)	1.39 (0.36–4.40, p = 0.597)	–	–	−21.58 (−32.60 to −10.57, p < 0.001) ^***^	−16.95 (−28.41 to −5.49, p = 0.004)
Tubular atrophy (yes or no)	2.05 (0.79–5.10, p = 0.127)	–	–	−4.62 (−13.70 to 4.45, p = 0.316)	–
Arterial intimal fibrosis (yes or no)	1.48 (0.62–3.48, p = 0.367)	–	–	−13.11 (−20.71 to −5.52, p = 0.001) ^**^	−9.68 (−17.53 to −1.84, p = 0.016)
Arteriolar hyalinosis (yes or no)	2.05 (0.79–5.10, p = 0.127)	–	–	−13.37 (−22.20 to −4.54, p = 0.003) ^**^	Excluded

DGF, delayed graft function; eGFR, estimated glomerular filtration rate.

^*^<0.05; ^**^<0.01; ^***^<0.001.

^#^The final model excluded the insignificant variables after adjustment.

We also evaluated the effect of chronic pathological changes (including glomerulosclerosis, interstitial fibrosis, tubular atrophy, arterial intimal fibrosis, and arteriolar hyalinosis) in the pretransplant biopsy on the allograft outcome (**Tables 5**, **6**). The existence of the chronic lesions except for tubular atrophy correlated with decreased posttransplant 1-year eGFR, while no effect was seen on the risk of DGF. After the confounding effect of the chronic pathological changes was adjusted, severe ATI was found to be not associated with 1-year eGFR.

**Table 6:** The chronic pathological lesions in pretransplant biopsy of grafts with severe ATI and mild ATI.

	All (140)	Mild ATI (122)	Severe ATI (18)	p-Value
Glomerulosclerosis, n (%)	37 (26.4)	32 (26.2)	5 (27.8)	1.000
Interstitial fibrosis, n (%)	16 (11.4)	14 (11.5)	2 (11.1)	1.000
Tubular atrophy, n (%)	30 (21.4)	28 (23.0)	2 (11.1)	0.362
Arterial intimal fibrosis, n (%)	45 (32.1)	41 (33.6)	4 (22.2)	0.487
Arteriolar hyalinosis, n (%)	30 (21.4)	28 (23.0)	2 (11.1)	0.362

ATI, acute tubular injury.

^*^<0.05; ^**^<0.01; ^***^<0.001.

### Pathological Change After Kidney Transplantation

Nine of the patients with severe ATI underwent 13 postoperative allograft biopsies, and the biopsy was performed from 2 weeks to 1 month posttransplant in 9 cases, 2 months in 2 cases, and 6 months in 2 cases. The pathological manifestations associated with renal tubular injury were described as tubular epithelium flattening, denudement of TBM, tubular cell calcification, granular cast, and regenerative changes. From 2 weeks to 1 month posttransplant, there was some degree of recovery from renal tubular injury, with severe lesions still present (denudement of TBM 22.2%) (**Table 7**), and most patients had begun to show regenerative changes (77.8%). At 2 months posttransplant, renal tubular injury was still present, but severe lesions had disappeared. At 6 months posttransplant, manifestations of renal tubular injury were hardly seen.

**Table 7:** Histopathological change of renal tubule in severe tubular injury after kidney transplantation.

Tubular injury description	2 weeks to 1 month (9)	2 months (2)	6 months (2)
Tubular epithelium flattening	8 (88.9)	2 (100.0)	0 (0)
Denudement of TBM	2 (22.2)	0 (0)	0 (0)
Tubular cell calcification	2 (22.2)	2 (100.0)	0 (0)
Granular cast	5 (55.6)	0 (0)	0 (0)
Regenerative changes	7 (77.8)	1 (50.0)	0 (0)

TBM, tubular basement membrane.

## Discussion

In this study, we developed a pragmatic pathological grading criterion for ATI. Severe ATI compromised short-term posttransplantation graft function; severe ATI was associated with an increased DGF rate, prolonged DGF recovery time, and lower eGFR before 6 months posttransplant. However, the kidney allograft function at 1 year and beyond posttransplant and the graft survival was not related to the severity of ATI. It was noteworthy that the performance of the prediction model for DGF including both donor creatinine and ATI severity was the best among all prediction models.

Long-term graft kidney function was closely related to the preexisting chronic lesions of the donor kidneys (e.g., glomerulosclerosis, interstitial fibrosis, arterial intimal fibrosis, and arteriolar hyalinosis). By analyzing the biopsy results at different times posttransplant, we found that at 2–4 weeks posttransplant, the donor kidney transplanted in the recipient still showed significant tubular injury but started to recover as regenerative change occurred. By 6 months posttransplant, the manifestations of renal tubular injury were almost absent. This reflected that severe ATI can be successfully recovered, and thus renal function can gradually return to normal. These results suggested that donor kidneys with ATI and no chronic lesions for renal transplantation have a good long-term prognosis, which is similar to the findings of previous studies (18). However, patients with severe ATI require special attention for perioperative and postoperative follow-up management. Patients with severe ATI are at high risk of DGF and, more importantly, have a significantly longer recovery time from DGF (taking an average of 50 days), which not only prolongs the hospital stay but also increases the risk of multiple complications such as postoperative infection and rejection (39), increasing the financial and psychological burden on the patient, which can affect the long-term prognosis if not managed promptly.

Our results indicated that ATI was associated with DCD status and cold ischemia time. This finding may be explained by the mechanisms by which ATI develops. ATI in a donor kidney is primarily the result of hypoxic–ischemic damage and nephrotoxic insults (20, 21, 23). The major pathological changes due to hypoxic–ischemic damage are reflected in the tubules through a sequence of events: vacuolation, swelling of the epithelium, loss of the brush border and flattening of the epithelium, and eventually cell necrosis and exposure of the basal membrane. As such, DCD status and cold ischemia time, which are related to hypoxic–ischemic damage, were identified as risk factors for developing severe ATI. We did not find a relation between severe ATI and warm ischemia time. This might be explained by the relatively short mean warm ischemia time in our study sample. Our findings are consistent with those of Hall et al. (40).

Prior studies that have examined whether or not ATI was related to DGF have provided conflicting results. We identified several studies that focused on the association of DGF and ATI or ATN (5, 28, 29, 31, 33, 35). Of the 6 studies, 3 found an association between ATN/ATI and DGF, and 3 did not find an association. An important explanation is that the definition of ATI/ATN varied among the studies (36). For example, a multicenter study by Hall et al. (33) with the largest sample size (N = 651) reveals no significant associations overall between preimplant biopsy-reported ATN and the outcomes of DGF. In the study, Hall et al. (33) classified all degrees of ATI as ATN, which would dilute the impact of severe ATI. In addition, 541 of the 651 grafts were considered to have normal tubules on pretransplantation biopsies. However, in our sample, few grafts were reported to have entirely normal tubules—most of the grafts had at least vacuolization. Several other studies did not give a specific definition of ATI/ATN, and it was not possible to evaluate the impact of their definitions.

Assessment of the donor kidneys with donor terminal serum creatinine or model prediction scores could only distinguish whether DGF would occur, but was not well enough to distinguish whether DGF recovery was fast or slow. The relationship between ATI severity and DGF recovery time has been poorly studied in the past. We have previously found a correlation between DGF recovery time and poor prognoses such as rejection, infection, or kidney allograft failure (33). In the study, we found that severe ATI prolongs DGF recovery time. The tubules regenerate through cellular proliferation, migration, and subsequent hypertrophy of a new population of proximal tubule cells (41). This process takes time; thus, severe ATI influences short-term allograft function, but in the long-term, the proximal tubules regenerate and graft function normalizes.

Our results showed that pretransplantation biopsy could provide additional valuable information to the quality assessment of the donor kidneys. First, severe ATI combined with high donor terminal serum creatinine was associated with a higher risk of DGF occurrence and a longer recovery time from DGF compared with high donor terminal serum creatinine alone. The model predictive ability of combining either donor serum creatinine level or calculated DGF risk with ATI severity for DGF rate was also better than that of donor serum creatinine level or calculated DGF risk alone. ATI severity can well predict DGF recovery time, while donor serum creatinine level or calculated DGF risk is not that effective. Therefore, the combination of clinical and pathological information allows for a comprehensive assessment of AKI in the donor kidneys. Second, a part of donor kidneys with low terminal serum creatinine will have severe tubular damage, possibly due to other factors such as ischemia during the process of dying, interventions during donor procurement, and damage during organ transportation and preservation. Acute injury to donor kidneys may not be identified in these donors if a pretransplant biopsy is not performed. Third, the chronic lesions in the donor kidneys could be evaluated in the biopsy, which were associated with the long-term allograft function in our study and prior studies (24). Therefore, it is necessary to perform a pretransplant renal allograft biopsy to clarify the severity of the ATI and the chronic lesions, especially for donors with high serum creatinine, DCD donors, or donors with long cold ischemia time.

We chose extremely and diffusely flattened renal tubular epithelial cells or denudement of TBM when developing the criteria for ATI severity for the following reasons. First, these manifestations can be easily identified in the frozen section. Second, a quick diagnosis is required in pretransplant donor kidney biopsy, so the simpler and more practical the criteria, the better. Third, a previous study on the relationship between different ATI pathological descriptions and the severity of clinical AKI found that only simplification (defined as flattened tubular cell cytoplasm with complete loss of brush border), mitosis, and cell sloughing (defined as a free-floating cell in the tubular lumen without attachment to adjacent cells or basement membrane) were able to independently predict severe AKI (42). This was consistent with our criteria. Mitosis was less frequent in the early stage of acute kidney injury and thus was not included in our criteria.

In our cohort, severe ATI appeared slightly worse in long-term eGFR compared to mild ATI, but not statistically significant. Kidney allograft fibrosis is an important cause of late graft loss, and previous studies suggest that ATI may be associated with renal fibrosis by combined mechanisms (43). Whether severe ATI can lead to deterioration of long-term renal allograft function by promoting renal fibrosis remains unanswered. Few clinical studies were found to explore the relationship between ATI and renal fibrosis in the background of kidney transplantation.

This analysis is limited by its retrospective nature and may be subject to selection bias, as the choice of donor kidney biopsy before transplantation was subjective and at the surgeon’s discretion.

In summary, based on our pragmatic dichotomous (mild-to-severe) grading criterion for ATI in a preimplantation biopsy, kidney allograft with severe ATI increased the risk of DGF, prolonged the DGF recovery time, and decreased the short-term graft function but demonstrated favorable long-term graft function. ATI severity in combination with donor terminal serum creatinine level performs better in predicting DGF occurrence compared with either ATI severity or donor terminal serum creatinine level alone. Pretransplant biopsy offered additive and valuable information in the assessment of AKI in the donor kidneys.

## Data Availability Statement

The raw data supporting the conclusions of this article will be made available by the authors, without undue reservation.

## Ethics Statement

The studies involving human participants were reviewed and approved by the Institutional Review Board of the First Affiliated Hospital of Sun-Yat Sen University. Written informed consent for participation was not required for this study in accordance with the national legislation and the institutional requirements.

## Author Contributions

JW, JQL, WW, and HZ designed the study, analyzed the data, and co-wrote the paper. SY and WC assessed the biopsies. LL, QF, JL, RD, CW, and WZ: performed data acquisition and interpretation. HZ, WW, XC, and SL performed the statistical analyses. HZ, HM, and WC supervised the research and critically reviewed it. All authors listed have made a substantial, direct, and intellectual contribution to the work and approved it for publication.

## Funding

This study was supported by the Science and Technology Planning Project of Guangdong Province, China (2015B020226002, 2017A020215012), National Natural Science Foundation of China (81870511, 82072824, 81772701), Key Scientific and Technological Program of Guangzhou City (201803040011), Guangdong Basic and Applied Basic Research Foundation (2020A1515010884), Guangdong Natural Science Foundation (2018A030313016), Clinical Research 5010 Programme of Sun Yat-Sen University, Guangdong Provincial Key Laboratory on Organ Donation and Transplant Immunology (2017B030314018, 2020B1212060026), and Guangdong Provincial International Cooperation Base of Science and Technology (Organ Transplantation, 2020A0505020003).

## Conflict of Interest

The authors declare that the research was conducted in the absence of any commercial or financial relationships that could be construed as a potential conflict of interest.

## Publisher’s Note

All claims expressed in this article are solely those of the authors and do not necessarily represent those of their affiliated organizations, or those of the publisher, the editors and the reviewers. Any product that may be evaluated in this article, or claim that may be made by its manufacturer, is not guaranteed or endorsed by the publisher.
